# Influence of the Type of Physician on Survival from Emergency-Medical-Service-Witnessed Cardiac Arrest: An Observational Study

**DOI:** 10.3390/healthcare10101841

**Published:** 2022-09-22

**Authors:** Miguel Freire-Tellado, Rubén Navarro-Patón, Javier Mateos-Lorenzo, Gabina Pérez-López, María del Pilar Pavón-Prieto, Marcos Mecías-Calvo

**Affiliations:** 1 Emergency Medical Services, Fundación Pública Urxencias Sanitarias de Galicia-061, 27001 Lugo, Spain; 2 Facultad de Formación del Profesorado, Universidade de Santiago de Compostela, 27001 Lugo, Spain; 3 Cantabrian Health Service 061, Nursion Division, 39770 Laredo, Spain; 4 Centro Integrado de Atención a las Emergencias, Fundación Pública Urxencias Sanitarias de Galicia-061, 36680 A Estrada, Spain; 5 Emergency Medical Services, Fundación Pública Urxencias Sanitarias de Galicia-061, 32005 Ourense, Spain

**Keywords:** OHCA, emergency-medical-service-witnessed cardiac arrest, prehospital physicians

## Abstract

Out-of-hospital cardiac arrest resuscitation by non-emergency dedicated physicians may not be positively associated with survival, as these physicians have less experience and exposure than specialised dedicated personnel. The aim of this study was to compare the survival results of the teams led by emergency dedicated physicians (EDPhy) with those of the teams led by non-emergency dedicated physicians (N-EDPhy) and with a team of basic life support (BLS) emergency technicians (EMTs) used as the control group. A retrospective, multicentre study of emergency-medical-service-witnessed cardiac arrest from medical causes in adults was performed. The records from 2006 to 2016 in a database of a regional emergency system were analysed and updated up to 31 December 2021. Two groups were studied: initial shockable and non-shockable rhythms. In total, 1359 resuscitation attempts were analysed, 281 of which belonged to the shockable group, and 1077 belonged to the non-shockable rhythm group. Any onsite return of spontaneous circulation, patients admitted to the hospital alive, global survival, and survival with a cerebral performance category (CPC) of 1-2 (good and moderate cerebral performance) were studied, with both of the latter categories considered at 30 days, 1 year (primary outcome), and 5 years. The shockable and non-shockable rhythm group (and CPC 1-2) survivals at 1 year were, respectively, as follows: EDPhy, 66.7 % (63.4%) and 14.0% (12.3%); N-EDPhy, 16.0% (16.0%) and 1.96 % (1.47%); and EMTs 32.0% (29.7%) and 1.3% (0.84%). The crude ORs were EDPhy vs. N-EDPhy, 10.50 (5.67) and 8.16 (4.63) (all *p <* 0.05); EDPhy vs. EMTs, 4.25 (2.65) and 12.86 (7.80) (*p <* 0.05); and N-EDPhy vs. EMTs, 0.50 (0.76) (*p <* 0.05) and 1.56 (1.32) (*p >* 0.05). The presence of an EDPhy was positively related to all the survival and CPC rates.

## 1. Introduction

Out-of-hospital cardiac arrest (OHCA) is a major health problem worldwide [1], and its survival results are poor [1], despite the various improvement strategies carried out by the emergency medical services (EMS) in various countries. Among these strategies, the presence of a physician as a team leader has been proposed, but the influence of the physician on the survival of OHCA remains a controversial subject, with studies [2,3,4,5,6,7,8] showing positive, inconclusive, or even negative results [9].

Emergency systems have been implemented based on scientific evidence [10,11], the availability of financial means, and the regulation of health personnel certifications. In Spain, as in other European countries, emergency services are based on a model led by a physician [8,12], and since there is no specialty in emergency medicine, emergency personnel are mainly composed of family medicine specialists and general practitioners.

The difficulty in guaranteeing assistance by emergency dedicated physicians to all types of emergencies of the population has led some EMS to send non-emergency dedicated physicians who are performing primary care consultation tasks to complete the teams of basic life support (BLS) emergency technicians to provide advanced life support (ALS); however, despite sharing specialty and the initial advanced life support training, emergency care is only a small part of the practice of these physicians and, therefore, could lead to less experience [6,13,14] and exposure to OHCA [6,15,16,17,18] and negatively affect the survival results.

The objective of the present study is to assess the influence of the type of physician who leads the resuscitation attempt on the survival from emergency-medical-service-witnessed cardiac arrest by comparing the survival results of emergency dedicated physicians (EDPhy) and non-emergency dedicated physicians (N-EDPhy). A BLS emergency medical technician (EMT) team was used as a control group to evaluate the influence of both types of medical leadership.

The primary outcome was global survival and survival with a cerebral performance category (CPC) of 1-2 (good and moderate cerebral performance) after 1 year, and the secondary outcomes were any onsite return of spontaneous circulation, patients admitted to the hospital alive, global survival, and CPC 1-2 survival at 30 days and 5 years. The shockable and non-shockable rhythms were studied independently.

The working hypothesis was that the presence of a physician dedicated exclusively to emergencies (EDPhy) leading the resuscitation attempts could have a positive relationship with survival.

## 2. Materials and Methods

### 2.1. Study Design, Sample, and Setting

This is a multicentre, descriptive, observational study with retrospective data collection from the OHCA database of the EMS “FPUS 061 of Galicia” adapted to the Utsein 2015 style [19]. The events from 2006 to 2016 inclusive were studied, and the survival rates were updated up to 31 December 2021. The data were encrypted and finally anonymised for handling. The request for informed consent was waived, and the study was approved by the Bioethics Committee of the Health Commission of Galicia, with registration number 2020/393, respecting the ethical principles of the Helsinki Convention.

The study was specifically designed to compare the influence of the team initially attending OHCAs with the fewest possible confounding variables that could influence the results (e.g., bystander influence, location, and response times). The aim was to evaluate the efficacy, i.e., the performance in the best possible conditions (EMS-witnessed VF only) of each part of the EMS.

The initial choice of the teams was made by the emergency centre 061 in response to the demands of urgent healthcare by the population, depending on availability and severity and assigned by the dispatcher. The assigned team arrived at the site and began patient assistance; at this time, all the patients were still alive. From this moment, the event to be studied was a cardiac arrest that occurred by chance and was witnessed by the healthcare provider team, who would then immediately initiate a resuscitation attempt. The surviving patients were transferred to various hospitals in the public network. Only those services requested by the general population were included, excluding healthcare locations and hospitals.

### 2.2. Description of Prehospital Emergency Medical Services in the Region

Each of the 18 autonomous regions in Spain has its own Public Health and Emergency Medical System (EMS). As there is no specialty in emergencies, the physicians who make up the emergency medical systems are mostly specialists in family medicine. All family physicians receive at least one certified advanced life support course and work one 17 to 24 h shift a week in an accident and emergency department for three years during their specialisation period. After concluding their specialty, every physician can voluntarily apply for an advanced life support refresher course once or twice a year.

The regional emergency system in the study period was composed of an emergency centre with a specific health phone number (061) and the 112 number for common general emergencies. The system consisted of 103 basic life support (BLS) ambulances with 2 technicians using automated external defibrillators, 10 advanced life support (ALS) ambulances, and 2 helicopters with physicians and nurses. When they were available, these ambulances with emergency dedicated physicians on board were the first option for being sent to the most critical patients, but the shortage of ALS resources caused the EMS to send primary care physicians and nurses as well when they were required to lead a BLS team to ensure ALS. As the EMS 061 was set up in July 1997, the EDPhy working for the EMS were younger and had fewer years of working experience within the period of the study than the N-EDPhy.

### 2.3. Study Population

The study was carried out in the Galicia Region of Spain, 49,500 km^2^ in area, with a gross domestic product below the Spanish average and an average population during the years of the study of approximately 2.7 million inhabitants. The region is very dispersed, with 7 urban centres with a population ranging from approximately 100,000 to 500,000 inhabitants and 313 municipalities.

### 2.4. Inclusion Criteria

Only cardiac arrests witnessed by emergency health personnel were included. OHCAs detected at EMS arrival were excluded. Only cardiac arrests with medical causes were included (medical causes include those cases in which the cause of cardiac arrest is presumed to be of cardiac origin, other medical causes, and those in which there is no obvious cause of cardiac arrest) [19]. Causes related to trauma, drug overdose, drowning, electrocution, and the external causes of asphyxia were excluded. Only patients 18 years or older were included. Futile CPR attempts (documented important comorbidities) were not included [20]. The episodes from internal cardioverter–defibrillator devices were not included. Teams comprising 4 members were compared (2 technicians, 1 physician, and 1 nurse), except for the EMT control group (2 technicians).

### 2.5. Study Variables

The main dependent variable to study was survival after an event (a cardiac arrest witnessed by emergency personnel) at the site of care (any return of spontaneous circulation at the site), after hospital admission (event survival), and at 30 days, 1 year, and 5 years after the event. The CPC at 30 days, 1 year, and 5 years were also studied.

As independent variables, the initial rhythm (shockable and non-shockable), and the team that performed the resuscitation (EDPhy, N-EDPhy, and EMTs) were considered.

### 2.6. Data Analysis

The characteristics of the patient groups are presented in the frequency tables as a percentage of the categorical data (i.e., gender), and with the median values with interquartile ranges for the continuous data (i.e., age) (Table 1). To compare the differences between the groups, bilateral tests were used. The categorical variables were analysed with the chi-square test, and the continuous variables were analysed with the Wilcoxon rank test. In this way, the association between exposure and outcome was examined. The associations are presented as odds ratios (ORs) with 95% confidence intervals (CIs).

Logistic regression was used to assess the trends in the participation of a type of emergency team with survival over time, with the initial rhythm, gender, and age range. Crude logistic regression was used to examine the association between exposure and outcome (survival) at each time point (i.e., any ROSC, event survival, 30-day, 1-year, and 5-year survival). The associations are represented as odds ratios (ORs) with 95% confidence intervals (CIs).

The first model was a survival analysis (yes; no) with the “enter” method, using the type of emergency medical service (EDPhy; N-EDPhy; EMTs); gender (male; female); age range (<65 years old; 66–75 years old; >76 years old); and the initial rhythm (shockable; non-shockable) as the explanatory covariates. To this end, all the above variables were entered as categorical, using the type of SEM as the first categorical reference variable at each of the moments studied (any ROSC, event survival, survival at 30 days, at one year, and at five years). The second explanatory model was obtained using the same covariates as in the first model but without taking into account the EDPhy medical service.

The Hosmer–Lemeshow goodness-of-fit test was used to determine whether the predicted probabilities deviated from the observed probabilities in a way that the binomial distribution did not predict.

All the data were processed using the SPSS version 24 statistical package for MS Windows (SPSS Inc., IBM Corporation, New York, NY, USA).

## 3. Results

### 3.1. Baseline Characteristics

**Figure 1:**
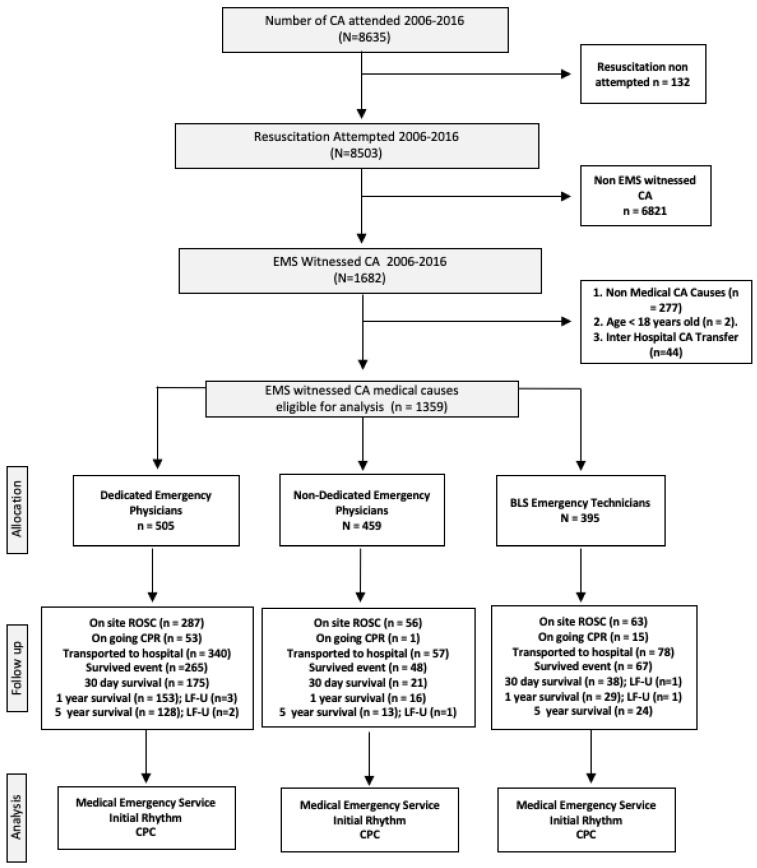
Flowchart. Note: EMS: emergency medical system; CA: cardiac arrest; ROSC: return of spontaneous circulation; CPR: cardiopulmonary resuscitation LF-U: lost to follow-up.

A total of 8503 resuscitation attempts were carried out during the period studied (2006–2016) (Figure 1). Excluded from this total number were 6821 OHCAs occurring previous to the EMS arrival, 277 OHCAs with non-medical causes (trauma, drug overdose, etc.), 44 witnessed in inter-hospital transfers (considered in-hospital cardiac arrest), and 2 cases of patients under 18 years old. The final sample for the study was 281 patients with shockable rhythms, comprising 156 patients treated by EDPhy, 50 by N-EDPhy, and 75 by EMTs (Table 1), and 1077 patients with non-shockable initial rhythms. The survival data were not obtained in four cases for survival at one year and another three cases for survival at five years due to private health insurance reasons (four cases), residence in another region (one case), and in another country (two cases).

The data results (Table 1) were disaggregated into two categories, namely the “initial shockable rhythms” and “initial non-shockable rhythms”, to avoid the influence of a disproportionate distribution.

### 3.2. Initial Shockable Rhythms

#### 3.2.1. Global Survival Rates

**Figure 2:**
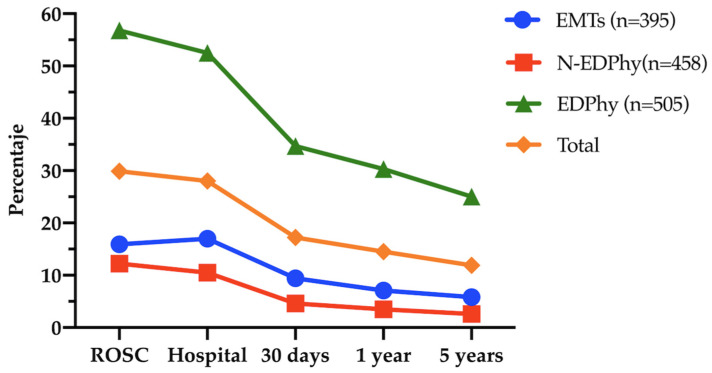
Survival percentage according to the type of medical emergency services. **Note:** EMTs: BLS emergency medical technicians; N-EDPhy: non-emergency dedicated physicians; EDPhy: emergency dedicated physicians.

Table 2 shows that during the period of time studied, all the survival results (any onsite recovery, patients admitted to the hospital alive, survival at 30 days, at 1 year, and 5 years) were significantly higher when the resuscitation was carried out by a team led by EDPhy than when led by the N-EDPhy group. Being treated by a team led by EDPhy meant having 11.80 times more likelihood of being admitted to the hospital alive when compared with treatment by the N-EDPhy team, and 8.83 times more likelihood of being alive at 5 years. When compared with the control EMT group, the presence of an EDPhy was associated with a likelihood of 4.74 times higher of being admitted to the hospital, whereas the presence of an N-EDPhy was associated with a decreased likelihood of survival (OR 0.54). The total survival percentage (for shockable and non-shockable initial rhythms) was higher for the attempt led by the EDPhy team and lower for that led by the N-EDPhy team (Figure 2). Overall, the initial rhythm was shockable in 20.7% of the cases, representing 63% of patients alive at 30 days and 75.1% at 5 years.

#### 3.2.2. CPC 1-2 Survival Rates

The incorporation of an EDPhy was positively associated with all the overall survival results (30 days, 1 year, and 5 years) with CPC 1-2 when compared with the N-EDPhy and EMT groups. The presence of an N-EDPhy was negatively related to the 30-day and 1-year survival results when compared with the EMT group (Table 3).

### 3.3. Initial Non-Shockable Rhythms

#### 3.3.1. Global Survival Rates

Table 2 shows that during the period of time studied, all the survival results (any onsite recovery, patients admitted to the hospital alive, survival at 30 days, and at 1 year, and 5 years) were significantly higher when the resuscitation was carried out by a team led by EDPhy than when led by the N-EDPhy group. When compared with the control EMT group, the presence of an EDPhy was significantly associated with all the survival rates, whereas the presence of an N-EDPhy was not significantly associated with the survival rates, with a small increased likelihood of survival (OR 0.83).

#### 3.3.2. CPC 1-2 Survival Rates

The incorporation of EDPhy was positively and significantly associated with all the overall survival results with CPC 1-2 when compared with N-EDPhy and EMT groups. The presence of N-EDPhy was non-significantly associated with all the survival results compared with the EMT group (Table 3).

### 3.4. Explanatory Logistic Regression Model

After applying a logistic regression model (Table 4), the participation of the EDPhy team was significantly positively associated with ROSC at the site (OR = 11.36), the event survival (OR = 9.48), and survival at 30 days (OR = 12.07), at 1 year (OR = 14.37) and at 5 years (OR = 11.66) when compared with the other groups, a relationship not dependent on the other confounding factors.

In a second predictive model, the participation of EMTs, compared with N-EDPhy, was significantly positively associated with all the survival results in the group <65 years and the event survival group (OR = 1.71) and with survival at 30 days (OR = 1.91) and at 1 year (OR = 1.88) in the overall results but did not reach statistical significance in the rest of the groups.

## 4. Discussion

The present study shows that the presence of a physician dedicated exclusively to emergencies was significantly related to greater survival results of the cardiac arrest witnessed by emergency personnel.

When compared with the EMT control group, the ALS provided by an EDPhy improved all the survival results, whereas the presence of an N-EDPhy did not significantly improve the results, and even worsened some survival results.

The overall and EDPhy survival rates are in the range of those published in the studies on the OHCAs witnessed by emergency personnel [21,22,23,24,25,26,27] in which shockable rhythms were the ones associated with greater survival and constituted the majority of living patients. These results were also consistent with the theoretical percentages expected for shockable rhythms proposed in the survival models [28]. However, the survival results of the N-EDPhy group were disturbing: Adding two members to provide ALS to an established EMT team did not improve and even decreased the survival results, contradicting the predicted results of the survival model [27], and the current knowledge about the advanced and basic life support [1]. To check this ALS-negative relationship with survival, we carried out research on the scientific literature, resulting in a paucity of information. With respect to general practitioners’ performance in cardiac arrest, several publications questioned the efficacy [28]. Another publication [29] analysing the survival rates of shockable rhythms obtained from a database of an OHCA registry of a European country revealed rates in a similar range to those found in the present study, although in other self-reported case studies, the results of survival rates were higher [30,31].

The results of this study show that specialisation works in emergencies, as in other fields of medicine. Thus, specific dedication to emergencies was positively associated with survival, meaning that specialisation works in improving survival rates, and this association is probably related to greater exposure to OHCA and experience [6,13,14,15,16,17,18]. This may call for the creation of a specialty in emergency medicine.

On the other hand, the ALS provided by specific emergency personnel should be extended to the largest possible proportion of the population served by the EMS. ALS did not always improve the survival rates of BLS. A benefit in terms of survival was added to BLS performance when ALS was provided by emergency dedicated personnel but not when it was delivered by N-EDPhy. Should only EDPhy be trained in ALS? Should immediate life support with easier recommendations be taught to N-EDPhy? [32] This disturbing fact is probably related to multiple factors such as a lack of exposure, training problems, and ALS protocols being too complicated, but above all, it might be indicative of a greater problem: Primary healthcare systems are already overwhelmed. Attending the emergency personnel causes physicians to abandon their patients in surgery and return later to deal with an increased number of waiting patients [32]. This stressful situation might weaken not only resuscitation attempts but also consultancy work.

Not all the initiatives adopted by the EMS with the objective of guaranteeing the best assistance translate into positive survival results [9,13,33], which shows the need for careful selection and a continuous evaluation of the strategies to obtain the best results for the treated population. Reporting the results, even when they are not as good as expected, is crucial to improvement (acting on the call).

The strength of this study is that it was specifically designed to compare team performance. By including only cardiac arrests in shockable rhythms witnessed by emergency medical personnel, the influence of multiple confounding variables dependent on time (arrival time), bystander (bystander CPR), the availability of automated external defibrillators, location (access to the scene), and the percentage of each type of initial rhythm was avoided. In this way, it was possible to compare team performance from the time zero of the event, and since the event was presented by chance once the team was at the scene and was not known by the dispatcher beforehand (randomly assigned), a lower influence of the remaining confounding variables was expected. The cerebral performance category and long-term survival were also studied.

Regarding the limitations, we should bear in mind that this was not a randomised study. In addition, the process of eliminating the potential confounding variables prior to team assistance resulted in a great reduction in the sample size. On the other hand, the distribution of the remaining confounding variables was not homogeneous, resulting in a disproportion of sex and age. This age disproportion might be explained by the fact that EDPhy were more experienced and could select their resuscitation attempts. It is also necessary to take into account that the model only evaluated the performance of the EMS personnel directly attending to the patient only. In spite of having excluded futile resuscitation attempts, previous medical conditions were not studied, and this could also affect the results. Although the event to be studied was the first 10 min of resuscitation by a team, other EDPhy resources could later arrive for post-resuscitation care or even to continue the resuscitation. It is also worth noting that, before being transferred to tertiary hospitals, the patients were initially transported to public hospitals with different levels of post-resuscitation care that could be at lower levels in those areas with small populations. However, the fact that the benefit in survival was greater in the earlier rates might suggest that the hospital effect was minimal [34]. Lastly, the teams led by the physicians had four members, while the EMT team used as the control group consisted of only two, which could also be related to survival [35,36,37].

## 5. Conclusions

The presence of a physician dedicated exclusively to emergencies as a leader of the resuscitation team significantly improved the survival results of the cardiac arrests witnessed by emergency personnel compared with those witnessed by the teams of primary care physicians.

Advanced life support improved the survival results of the basic life support control group when it was provided by an emergency dedicated physician but not when it was delivered by a non-emergency dedicated physician.

## Figures and Tables

**Table 1:** Total attended rhythms.

Variable	Global	Emergency Dedicated Physicians	Non-Emergency Dedicated Physicians	BLS Emergency Medical Technicians	*p*-Value
**Study population (n, %)**	1359 (100)	505 (37.2)	458 (33.7)	395 (29.1)	0.001
**Sex, (n, %)**					
Male	875 (64.4)	361 (41.3)	275 (31.4)	239 (27.3)	>0.005
Female	483 (35.6)	144 (29.8)	183 (37.9)	156 (32.3)	>0.005
**Age, median (IQR)**	70.03 (22–95)	66.82 (22–95)	72.86 (29–95)	70.85 (26–94)	<0.001
<65 years old (n,%)	421 (31.0)	201 (47.7)	109 (25.9)	111 (26.4)	<0.001
From 66 to 75 years old (n,%)	367 (27.0)	144 (39.2)	115 (31.3)	108 (29.4)	< 0.001
>76 years old (n,%)	570 (42.0)	160 (28.1)	234 (41.1)	176 (30.9)	< 0.001
**First rhythm Shockable (n, %)**	281 (20.7)	156 (30.9)	50 (10.9)	75 (19.0)	>0.005
**Non-shockable (n, %)**					
Asystole	402 (29.6)	128 (25.3)	146 (31.9)	128 (32.4)	>0.005
Undefined	64 (4.7)	0 (0.0)	39 (8.5)	25 (6.3)	>0.005
PEA	611 (45.0)	221 (43.8)	223 (48.7)	167 (42.3)	>0.005

**Table 2:** Survival of the sample (any ROSC, upon arrival at the hospital, one month, one year, and five years after the event based on the EMS that attends it and the initial rhythm).

	EMS (n)	ROSC (n, %)	Hospital (n, %)	30 Days (n, %)	1 Year (n, %)	5 Years (n, %)
**Total**	(1) EMTs (n = 395)	63 (15.9)	67 (17.0)	37 (9.4)	28 (7.1)	23 (5.8)
	(2) N-EDPhy (n = 458)	56 (12.2)	48 (10.5)	21 (4.6)	16 (3.5)	12 (2.6)
	(3) EDPhy (n = 505)	287 (56.8)	265 (52.5)	175 (34.7)	153 (30.3)	126 (25.0)
**Global**	n = 1358	406 (29.9)	380 (28.0)	233 (17.2)	197 (14.5)	161 (11.9)
	**Odds Ratio (CI)**					
	2 vs. 1	0.77 (0.54–1.07)	0.62 (0.44–0.87) **	0.49 (0.29–0.82) **	0.49 (0.27–0.89) ***	0.45 (0.23–0.89) ***
	3 vs. 1	8.75 (6.28–12.19) *	5.40 (3.94–7.41) *	5.15 (3.50–7.56) *	5.68 (3.70–8.72) *	5.38 (3.37–8.57) *
	3 vs. 2	12.45 (8.87–17.48) *	9.43 (6.67–13.33) *	11.06 (6.88–17.79) *	12.01-(7.04–20.47) *	12.36 (6.73–22.69) *
**Shockable Initial rhythm**	(1) EMTs (n = 75)	31 (41.3)	33 (44.0)	27 (36.0)	24 (32.0)	22 (29.3)
	(2) N-EDPhy (n = 50)	14 (28.0)	12 (24.0)	9 (18.0)	8 (16.0)	7 (14.0)
	(3)EDPhy (n = 156)	127 (81.4)	123 (78.8)	111 (71.6)	104 (66.7)	92 (59.0)
**Global**	n = 281	172 (61.2)	168 (59.8)	147 (52.5)	136 (48.4)	121 (43.1)
	**Odds Ratio (CI)**					
	2 vs. 1	0.67 (0.40–1,14) ***	0.54 (0.31–0.95) ***	0.50 (0.26–0.97) ***	0.50(0.24–1.02) ***	0.47 (0.22–1.03) ***
	3 vs. 1	6.947 (3.63–13.28) *	4.744 (2.61–8.61) *	4.485 (2.49–8.06) *	4.250 (2.36–7.65) *	3.46 (1.918–6.25) *
	3 vs. 2	14.199 (6.64–30.38) *	11.80 (5.52–25.09) *	11.49 (5.16–25.61) *	10.50 (4.60–23.99) *	8.83 (3.74–20.87) *
**Non-shockable Initial rhythm**	(1) EMTs (n = 320)	32 (10.0)	34 (10.6)	10 (3.1)	4 (1.3)	1 (0.3)
	(2) N-EDPhy (n = 408)	42 (10.3)	36 (8.82)	12 (2.94)	8 (1.96)	5 (1.22)
	(3) EDPhy (n = 349)	160 (45.8)	142 (40.7)	64 (18.3)	49 (14.0)	34 (9.7)
**Global**	n = 1077	234 (21.7)	212 (19.7)	86 (8.0)	61 (5.7)	40 (3.7)
	**Odds Ratio (CI)**					
	2 vs. 1	1.000 (0.62–1.63)	0.83 (0.53–1.29)	0.94 (0.41–2.15)	1.56 (0.47–5.14)	3.92 (0.46–33.39)
	3 vs. 1	9.789 (6.36–15.05) *	5.770 (3.81–8.74) *	6.961 (3.50–13.82) *	12.863 (4.59–36.08) *	34.432 (4.68–253.06) *
	3 vs. 2	9.792 (6.62–14.48) *	7.089 (4.74–10.61) *	7.411 (3.93–13.98 ) *	8.167(3.81–17.50) *	8.700 (3.64–22.50) *

Note: EMTs: basic life support emergency medical technicians; N-EDPhy: non-emergency dedicated physicians; EDPhy: emergency dedicated Physicians; * = *p <* 0.001; ** = *p <* 0.005; *** *p <* 0.05.

**Table 3:** Survival of the sample and cerebral performance category (CPC, 1-2) of the event based on the EMS that attends it and the initial rhythm.

		EDPhy (1) (n, %)	N-EDPhy (2) (n, %)	EMTs (3) (n, %)	*p*-Value	Odds Ratio (CI)	
**Global**	Total resuscitations	505 (37.2)	458 (33.7)	395 (29.1)	0.001	-	
	Overall survival at 30 days with CPC	160 (31.68)	18 (3.93)	29 (7.34)	<0.001	**1 vs. 2**	5.54 (3.56–8.62)
						**1 vs. 3**	3.35 (2.38–4.72)
						**2 vs. 3**	0.736 (0.580–0.93)
	Overall survival at 1 year with CPC	142 (27.97)	14 (3.06)	25 (6.32)	<0.001	**1 vs. 2**	6.13 (3.70–10.14)
						**1 vs. 3**	3.37 (2.33–4.87)
						**2 vs. 3**	0.70 (0.55–0.90)
	Overall survival at 5 years with CPC	120 (24.95)	12 (2.62)	21 (5.82)	<0.001	**1 vs. 2**	5.90 (3.43–10.16)
						**1 vs. 3**	3.38 (2.21–4.94)
						**2 vs. 3**	0.71 (0.54–0.93)
**Shockable Initial rhythm**	Total resuscitations	156 (55.51)	50 (17.79)	75 (26.69)	<0.001	-	
	Overall survival at 30 days with CPC	108 (69.23)	8 (16.0)	24 (32.0)	<0.001	**1 vs. 2**	6.76 (3.34–13.68)
						**1 vs. 3**	2.83 (1.88–4.26)
						**2 vs. 3**	0.73 (0.55–0.96)
	Overall survival at 1 year with CPC	99 (63.46)	8 (16.0)	22 (29.73)	<0.001	**1 vs. 2**	5.67 (2.80–11.48)
						**1 vs. 3**	2.65 (0.1.73–4.05)
						**2 vs. 3**	0.76 (0.57–1.00)
	Overall survival at 5 years with CPC	91 (58.33)	8 (16.0)	20 (26.66)	<0.001	**1 vs. 2**	4.85 (2.40–9.83)
						**1 vs. 3**	2.54 (1.63–3.95)
						**2 vs. 3**	0.79 (0.59–1.06)
	Total resuscitations	349 (32.40)	408 (37.88)	320 (29.71)	0.004	**-**	
**Non-shockable** **Initial rhythm**	Overall survival at 30 days with CPC	52 (14.89)	10 (2.45)	5 (1.56)	<0.001	**1 vs. 2**	3.55 (2.00–6.28)
						**1 vs. 3**	5.86 (2.53–13.60)
						**2 vs. 3**	1.32 (0.64–2.72)
	Overall survival at 1 year with CPC	43 (12.32)	6 (1.47)	3 (0.94)	<0.001	**1 vs. 2**	4.63 (2.18–9.83)
						**1 vs. 3**	7.80 (2.60–23.36)
						**2 vs. 3**	1.32 (0.52–3.34)
	Overall survival at 5 years with CPC	29 (8.30)	4 (0.98)	1 (0.31)	<0.001	**1 vs. 2**	4.60 (1.83–11.56)
						**1 vs. 3**	14.97 (2.17–103.03)
						**2 vs. 3**	2.20 (0.38–12.75)

Note: EMTs: basic life support emergency technicians; N-EDPhy: non-emergency dedicated physicians; EDPhy: emergency dedicated physicians; CPC: cerebral performance category good (1) and moderate (2).

**Table 4:** Survival regression models based on the types of variables, namely SEM, age range, gender, and initial rhythm.

					95% CI	
		Chi^2^ Wald	*p*-Value	Exp (B)	Low	Up
**Survival**	**Model 1**					
ROSC		197.61	<0.001	11.35	7.93	16.26
Hospital		176.09	<0.001	9.487	6.62	13.58
30 days	EDPhy	97.61	<0.001	12.07	7.01	18.36
1 year		81.41	<0.001	14.37	7.03	20.75
5 years		60.72	<0.001	11.66	6.29	21.62
	**Model 2**					
ROSC	EMTs	2.868	0.090	1.402	0.948	2.072
	<65 years old	7.086	0.008	1.572	1.127	2.193
Hospital	EMTs	7.024	0.008	1.716	1.151	2.558
	<65 years old	6.07	0.014	1.530	1.09	2.15
30 days	EMTs	4.95	0,026	1.91	1.08	3.37
	<65 years old	17.21	<0.001	2.30	1.55	3.41
1 year	EMTs	3.66	0.041	1.88	0.98	3.59
	<65 years old	18.30	<0.001	2.206	1.427	3.411
5 years	EMTs	3.580	0.058	2.061	0.974	4.358
	<65 years old	26.707	<0.001	3.800	2.290	6.305

Note: EDPhy: dedicated emergency physicians; EMTs: BLS emergency technicians.

## Data Availability

Restrictions apply to the availability of these data. The data were obtained from the Public Health Emergency Foundation 061 and are available to the authors with their authorisation.

## References

[1] Monsieurs K.G., Nolan J.P., Bossaert L.L., Greif R., Maconochie I.K., Nikolaou N.I., Perkins G.D., Soar J., Truhlář A., Wyllie J. (2015). European Resuscitation Council Guidelines for Resuscitation 2015: Section 1. Executive summary. Resuscitation.

[2] Hamilton A., Steinmetz J., Wissenberg M., Torp-Pedersen C., Lippert F.K., Hove L., Lohse N. (2016). Association between prehospital physician involvement and survival after out-of-hospital cardiac arrest: A Danish nationwide observational study. Resuscitation.

[3] Böttiger B.W., Grabner C., Bauer H., Bode C., Weber T., Motsch J., Martin E. (1999). Long term outcome after out-of-hospital cardiac arrest with physician staffed emergency medical services: The Utstein style applied to a midsized urban/suburban area. Heart.

[4] Böttiger B.W., Bernhard M., Knapp J., Nagele P. (2016). Influence of EMS-physician presence on survival after out-of-hospital cardiopulmonary resuscitation: Systematic review and meta-analysis. Crit. Care.

[5] Estner H.L., Günzel C., Ndrepepa G., William F., Blaumeiser D., Rupprecht B., Hessling G., Deisenhofer I., Weber M.A., Wilhelm K. (2007). Outcome after out-of-hospital cardiac arrest in a physician-staffed emergency medical system according to the Utstein style. Am. Heart J..

[6] Dyson K., Bray J., Smith K., Bernard S., Finn J. (2014). A systematic review of the effect of emergency medical service practitioners’ experience and exposure to out-of-hospital cardiac arrest on patient survival and procedural performance. Resuscitation.

[7] Von Vopelius-Feldt J., Coulter A., Benger J. (2015). The impact of a pre-hospital critical care team on survival from out-of-hospital cardiac arrest. Resuscitation.

[8] Fischer M., Kamp J., Garcia-Castrillo Riesgo L., Robertson-Steel I., Overton J., Ziemann A., Krafft T. (2011). Comparing emergency medical service systems—A project of the European Emergency Data (EED) Project. Resuscitation.

[9] Yen Z.-S., Chen Y.-T., Ko P.C.-I., Ma M.H.-M., Chen S.-C., Chen W.-J., Lin F.-Y. (2006). Cost-effectiveness of Different Advanced Life Support Providers for Victims of Out-of-hospital Cardiac Arrests. J. Formos. Med. Assoc..

[10] Bossaert L.L. (1993). The complexity of comparing different EMS systems—A survey of EMS systems in Europea. Ann. Emerg. Med..

[11] Fevang E., Lockey D., Thompson J., Lossius H.M., Collaboration T.T.R. (2011). The top five research priorities in physician-provided pre-hospital critical care: A consensus report from a European research collaboration. Scand. J. Trauma Resusc. Emerg. Med..

[12] Rosell-Ortiz F., Escalada-Roig X., Fernández del Valle P., Sánchez-Santos L., Navalpotro-Pascual J.M., Echarri-Sucunza A., Adsuar-Quesada J.M., Ceniceros-Rozalén I., Ruiz-Azpiazu J.I., Ibarguren-Olalde K. (2017). Out-of-hospital cardiac arrest (OHCA) attended by mobile emergency teams with a physician on board. Results of the Spanish OHCA Registry (OSHCAR). Resuscitation.

[13] Silfvast T., Ekstrand A. (1996). The effect of experience of on-site physicians on survival from prehospital cardiac arrest. Resuscitation.

[14] Bjornsson H.M., Marelsson S., Magnusson V., Sigurdsson G., Thorgeirsson G. (2011). Physician experience in addition to ACLS training does not significantly affect the outcome of prehospital cardiac arrest. Eur. J. Emerg. Med..

[15] Weiss N., Ross E., Cooley C., Polk J., Velasquez C., Harper S., Walrath B., Redman T., Mapp J., Wampler D. (2018). Does Experience Matter? Paramedic Cardiac Resuscitation Experience Effect on Out-of-Hospital Cardiac Arrest Outcomes. Prehospital Emerg. Care.

[16] Tuttle J.E., Hubble M.W. (2018). Paramedic Out-of-hospital Cardiac Arrest Case Volume Is a Predictor of Return of Spontaneous Circulation. West. J. Emerg. Med..

[17] Bray J., Nehme Z., Nguyen A., Lockey A., Finn J. (2020). A systematic review of the impact of emergency medical service practitioner experience and exposure to out of hospital cardiac arrest on patient outcomes. Resuscitation.

[18] Dyson K., Bray J.E., Smith K., Bernard S., Straney L., Finn J. (2016). Paramedic Exposure to Out-of-Hospital Cardiac Arrest Resuscitation Is Associated With Patient Survival. Circ. Cardiovasc. Qual. Outcomes.

[19] Perkins G.D., Jacobs I.G., Nadkarni V.M., Berg R.A., Bhanji F., Biarent D., Bossaert L.L., Brett S.J., Chamberlain D., de Caen A.R. (2015). Cardiac Arrest and Cardiopulmonary Resuscitation Outcome Reports: Update of the Utstein Resuscitation Registry Templates for Out-of-Hospital Cardiac Arrest: A Statement for Healthcare Professionals From a Task Force of the International Liaison Committee. Resuscitation.

[20] Einav S., Alon G., Kaufman N., Braunstein R., Carmel S., Varon J., Hersch M. (2012). To resuscitate or not to resuscitate: A logistic regression analysis of physician-related variables influencing the decision. Emerg. Med. J..

[21] Sasson C., Rogers M.A.M., Dahl J., Kellermann A.L. (2010). Predictors of Survival From Out-of-Hospital Cardiac Arrest. Circ. Cardiovasc. Qual. Outcomes.

[22] Hostler D., Thomas E.G., Emerson S.S., Christenson J., Stiell I.G., Rittenberger J.C., Gorman K.R., Bigham B.L., Callaway C.W., Vilke G.M. (2010). Increased survival after EMS witnessed cardiac arrest. Observations from the Resuscitation Outcomes Consortium (ROC) Epistry—Cardiac arrest. Resuscitation.

[23] Kajino K., Kitamura T., Kiyohara K., Iwami T., Daya M., Ong M.E.H., Shimazu T., Sadamitsu D. (2016). Temporal Trends in Outcomes after Out-of-Hospital Cardiac Arrests Witnessed by Emergency Medical Services in Japan: A Population-Based Study. Prehospital Emerg. Care.

[24] Lindner T.W., Søreide E., Nilsen O.B., Torunn M.W., Lossius H.M. (2011). Good outcome in every fourth resuscitation attempt is achievable—An Utstein template report from the Stavanger region. Resuscitation.

[25] Chia M.Y.C., Kwa T.P.W., Wah W., Yap S., Doctor N.E., Ng Y.Y., Mao D.R., Leong B.S.-H., Gan H.N., Tham L.P. (2019). Comparison of Outcomes and Characteristics of Emergency Medical Services (EMS)-Witnessed, Bystander-Witnessed, and Unwitnessed Out-of-Hospital Cardiac Arrests in Singapore. Prehospital Emerg. Care.

[26] Axelsson C., Claesson A., Engdahl J., Herlitz J., Hollenberg J., Lindqvist J., Rosenqvist M., Svensson L. (2012). Outcome after out-of-hospital cardiac arrest witnessed by EMS: Changes over time and factors of importance for outcome in Sweden. Resuscitation.

[27] Nehme Z., Andrew E., Bernard S., Smith K. (2016). Impact of cardiopulmonary resuscitation duration on survival from paramedic witnessed out-of-hospital cardiac arrests: An observational study. Resuscitation.

[28] Larsen M.P., Eisenberg M.S., Cummins R.O., Hallstrom A.P. (1993). Predicting survival from out-of-hospital cardiac arrest: A graphic model. Ann. Emerg. Med..

[29] Masterson S., Vellinga A., Wright P., Dowling J., Bury G., Murphy A.W. (2015). General practitioner contribution to out-of-hospital cardiac arrest outcome: A national registry study. Eur. J. Gen. Pract..

[30] Barry T., Headon M., Glynn R., Conroy N., Tobin H., Egan M., Bury G. (2018). Ten years of cardiac arrest resuscitation in Irish general practice. Resuscitation.

[31] Bury G., Headon M., Dixon M., Egan M. (2009). Cardiac arrest in Irish general practice: An observational study from 426 general practices. Resuscitation.

[32] Vinker S. (2017). Out of hospital Cardio-pulmonary arrest-Is there a role for the primary healthcare teams?. Isr. J. Health Policy Res..

[33] Stiell I.G., Wells G.A., Field B., Spaite D.W., Nesbitt L.P., De Maio V.J., Nichol G., Cousineau D., Blackburn J., Munkley D. (2004). Advanced Cardiac Life Support in Out-of-Hospital Cardiac Arrest. N. Engl. J. Med..

[34] Spaite D.W., Bobrow B.J., Stolz U., Berg R.A., Sanders A.B., Kern K.B., Chikani V., Humble W., Mullins T., Stapczynski J.S. (2014). Statewide Regionalization of Postarrest Care for Out-of-Hospital Cardiac Arrest: Association With Survival and Neurologic Outcome. Ann. Emerg. Med..

[35] Warren S.A., Prince D.K., Huszti E., Rea T.D., Fitzpatrick A.L., Andrusiek D.L., Darling S., Morrison L.J., Vilke G.M., Nichol G. (2015). Volume versus outcome: More emergency medical services personnel on-scene and increased survival after out-of-hospital cardiac arrest. Resuscitation.

[36] Sun J.-T., Chiang W.-C., Hsieh M.-J., Huang E.P.-C., Yang W.-S., Chien Y.-C., Wang Y.-C., Lee B.-C., Sim S.-S., Tsai K.-C. (2018). The effect of the number and level of emergency medical technicians on patient outcomes following out of hospital cardiac arrest in Taipei. Resuscitation.

[37] Ng Q.X., Han M.X., Lim Y.L., Arulanandam S. (2021). A Systematic Review and Meta-Analysis of the Implementation of High-Performance Cardiopulmonary Resuscitation on Out-of-Hospital Cardiac Arrest Outcomes. J. Clin. Med..

